# Well Water

**Published:** 1890-06

**Authors:** 

**Affiliations:** Rutgers College


### WELL WATER.

The average house owner certainly believes that the water which he pumps clear and cool from his well is pure and wholesome. He does not stop to think of the impurities with which it may have come in contact during its flow from the surface to the bottom of his well. This well may be sunk in the immediate vicinity of an overflowing cess-pool or out-house; the natural drainage of his own or his neighbor's barn-yard or pig-sty may be flowing over the soil through which is filtering the water that is to fill the underground cistern ; or its bottom may be in a porous stratum of soil or gravel that receives, at a point higher than the bottom, the drainage from some graveyard or other source of decaying organic matter ; some neighboring tree may have thrust its rootlets through the wall of the well and there they remain to decay ; or the top may not be tightly covered and strong, toads and other vermin may tumble in to aid in the pollution of the supply ; but our well owner, not seeing, smelling or tasting the results of these additions to the underground reservoir, is not conscious of their existence.

If the flow of organic impurities through a natural filter bed be so great as to fill it with this precipitated organic matter, decomposition of the masses of organic filth thus carried into the soil takes place, and the soluble products of this decomposition flow on with the underground streams until a well offers a collecting place for them. Nor is this all. The soil being taxed by the large amount of impurities sent through it beyond its filtering power, allows these soluble products to pass unchanged, and they are carried directly into the well, where the cessation of flow allows them to accumulate. Such a filth-saturated condition of the soil exists in every old and thickly settled community. Near every stable, every out-house or cess-pool, with their porous walled (if walled at all) vaults, every kitchen drain and sewer, is furnishing its quota of organic impurities, all of which supply matter for decomposition.

The products of this decomposition are carried, as we have seen, directly to the wells, and they thus become suitable breeding places for bacterial life—powder magazines, only needing the spark of a typhoid or other deadly germ to furnish the explosion of a scourge of disease. —*Prof. Wilbur*, *Rutgers College.*

